# Comparative Structural Analysis of Lipid Binding START Domains

**DOI:** 10.1371/journal.pone.0019521

**Published:** 2011-06-30

**Authors:** Ann-Gerd Thorsell, Wen Hwa Lee, Camilla Persson, Marina I. Siponen, Martina Nilsson, Robert D. Busam, Tetyana Kotenyova, Herwig Schüler, Lari Lehtiö

**Affiliations:** 1 Department of Medical Biochemistry and Biophysics, Structural Genomics Consortium, Karolinska Institutet, Stockholm, Sweden; 2 Structural Genomics Consortium, University of Oxford, Headington, Oxford, United Kingdom; 3 Department of Biosciences, Pharmaceutical Sciences, Åbo Akademi University, Turku, Finland; University of Cambridge, United Kingdom

## Abstract

**Background:**

Steroidogenic acute regulatory (StAR) protein related lipid transfer (START) domains are small globular modules that form a cavity where lipids and lipid hormones bind. These domains can transport ligands to facilitate lipid exchange between biological membranes, and they have been postulated to modulate the activity of other domains of the protein in response to ligand binding. More than a dozen human genes encode START domains, and several of them are implicated in a disease.

**Principal Findings:**

We report crystal structures of the human STARD1, STARD5, STARD13 and STARD14 lipid transfer domains. These represent four of the six functional classes of START domains.

**Significance:**

Sequence alignments based on these and previously reported crystal structures define the structural determinants of human START domains, both those related to structural framework and those involved in ligand specificity.

**Enhanced version:**

**This article can also be viewed as an enhanced version in which the text of the article is integrated with interactive 3D representations and animated transitions. Please note that a web plugin is required to access this enhanced functionality. Instructions for the installation and use of the web plugin are available in Text S1.**

## Introduction

The START domain is a ubiquitous conserved module for binding and transporting lipids [1]. Although the functions of most START domain containing proteins remain unknown, some regulate steroidogenesis and some are known to transfer lipids between membranes. There are approximately 40 proteins containing domains with START homology encoded in the human genome. The most well-characterized START domain containing proteins have been divided into 6 groups based on their phylogenetic relationships [2], [3], but additional members can be assigned to most of these groups. Group 1 contains the name-giving family member, steroidogenic acute regulatory protein (StAR/STARD1), and STARD3. Both are cholesterol carriers, and mutations in STARD1 cause congenital lipoid adrenal hyperplasia. Group 2 consist of proteins containing only a START domain; group 3 proteins are capable of binding different ligands, such as phosphatidyl choline (STARD2/PCTP) and ceramides (STARD11); group 4 proteins (DLC, or deleted in cancerous liver cells) are frequently de-regulated in cancer and contain Rho-GTPase activating domains; group 5 proteins contain two thioesterase domains; and group 6 consists of only STARD9, a 4614-residue protein with unknown function, that contains a kinesin motor domain at its N-terminus. Mitochondria contain at least the group 2 phosphatidylcholine transfer protein STARD7, and also the Coenzyme Q binding protein Coq10, which was recently identified to contain a divergent START domain [4].

Structural analyses of START domains from groups 1–3 have provided detailed insights into how these proteins sequester specific lipids [5]–[9] (summarized in Table 1). The ∼210 residue globular START module is a curved β-sheet gripped by two α-helices. The concave face of the β-sheet and the C-terminal α-helix enclose a hydrophobic cavity that can accommodate lipid molecules. Here we present crystal structures of four human START domains, those of STARD1, STARD5, STARD13 and STARD14/ACOT11. These structures extend our knowledge onto group 4 and 5 START domains, and enable a family-wide comparison of their lipid binding cavities. This structural comparison also sheds light on the lipid specificity of START proteins.

**Table 1:** Human START proteins, their ligands, and the available crystal structures.

Group	Protein	Ligand	PDB entry
**1 - StAR**	STARD1	cholesterol [5]	3P0L; ligand-free (this study)
	STARD3/MLN64	cholesterol [5]	1EM2; ligand-free [5]
**2 - START only**	STARD4	cholesterol [19]	1JSS (mouse); ligand-free [7]
	STARD5	cholesterol, 25-hydroxycholesterol [19]	2R55; ligand-free (this study)
	STARD6	cholesterol [40]	-
**3 - PCTP**	STARD2/PCTP	phosphatidyl choline [41]	1LN1; DLP complex^1^ [6]
	STARD7	phosphatidyl choline [42]	-
	STARD10	phosphatidylcholine/ethanolamine [43]	-
	STARD11/CERT	ceramides [8]	2E3R; C18-ceramide complex [8]; 2Z9Z; C10-DAG complex^2^ [8]; 3H3S; H15 complex^3^ [9] and 10 more entries
**4 - RhoGAP**	STARD8	charged lipid?	-
	STARD12	charged lipid?	-
	STARD13	charged lipid?	2PSO; ligand-free (this study)
**5 - Thioesterase**	STARD14	fatty acid?	3FO5; PEG complex^4^ (this study)
	STARD15	fatty acid?	-
**6 - STARD9**	STARD9	?	-

1 DLP, 1,2-dilinoleoyl-SN-glycero-3-phosphocholine.

2 DAG, diacylglycerol.

3 H15, 3-hydroxy-1-(hydroxymethyl)-3- phenylpropyl]pentadecanamide.

4 PEG, pentaethylene glycol.

## Results

We used a structural genomics approach to human START domain containing proteins. Based on previously published crystal structures multiple expression constructs were designed for STARD1, STARD5, STARD7–11, STARD13 and STARD14. Following recombinant protein production in *E. coli*, well-diffracting crystals were obtained for the START domains of STARD1, STARD5, STARD13 and STARD14 (Table 2). Their crystal structures were solved and refined to between 2.0 and 3.4 Å resolution (Table 3).

**Table 2:** Summary of crystallization and cryo conditions.

Condition	STARD1native	STARD5native	STARD13native	STARD14native	STARD14SeMet
**Salt**	200 mM Ca-acetate	-	125 mM NaCl	200 mM MgCl_2_	200 mM NaSCN
**Precipitant**	40% PEG-300	10% PEG-6000	20% MPD	25% PEG-3350	20% PEG-3350
**Buffer**	100 mM Na-cacodylate	100 mM HEPES	100 mM Tris-HCl	100 mM Bis-Tris	
**pH**	6.5	7.0	8.0	5.5	6.9
**Additive**	Cholesterol	-	-	-	-
**Temperature**	4°C	4°C	4°C	4°C	4°C
**Method**	Sitting drop	Sitting drop	Hanging drop	Sitting drop	Sitting drop
**Drop size (protein/well solution) (**µl)**	0.2/0.2	0.8/0.4	0.8/0.2	0.2/0.2	0.2/0.4
**Cryo additive**	50% PEG-300	20% BD	40% MPD	20% Glycerol	18% Glycerol

**Table 3:** Data collection statistics.*

Protein	STARD1	STARD5	STARD13	STARD14	STARD14 SeMet pk	STARD14 SeMet ip	STARD14 SeMet rm
**X-ray source**	ESRF ID29	ESRF ID14.4	ESRF ID14.4	ESRF ID14-2	BESSY BL14-2	BESSY BL14-2	BESSY BL14-2
**Wavelength (Å)**	0.97948	1.03992	1.03992	0.93300	0.97973	0.97985	0.97201
**Space group**	P6_3_	P6_5_	P4_3_	C222_1_	P3_1_	P3_1_	P3_1_
**Cell dimensions**							
***a*** **, ** ***b*** **, ** ***c*** ** (Å)**	144.130, 144.130, 101.030	62.87, 62.87, 214.93	78.24, 78.24, 212.72	52.44, 130.08, 165.23	69.41, 69.41, 97.25	69.41, 69.41, 97.25	69.41, 69.41, 97.25
***α*** **, ** ***β*** **, ** ***γ*** ** (°)**	90, 90, 120	90, 90, 120	90, 90, 90	90, 90, 90	90, 90, 120	90, 90, 120	90, 90, 120
**Resolution (Å)**	15-3.4 (3.5-3.4)	20-2.5 (2.6-2.5)	20-2.8 (2.9-2.8)	20-2.0 (2.1-2.0)	50-2.3 (2.36-2.3)	50-2.3 (2.36-2.3)	50-2.3 (2.36-2.3)
**R_merge_** †	0.173 (0.797)	0.102 (0.671)	0.038 (0.556)	0.055 (0.545)	0.040 (0.468)	0.037 (0.471)	0.036 (0.533)
**I/(σI)**	19.8 (4.8)	23.3 (5.9)	15.2 (3.7)	15.3 (2.6)	10.1 (1.6)	10.8 (1.6)	11.5 (1.4)
**Completeness (%)**	98.6 (100)	99.4 (99.5)	99.5	98.1 (99.5)	98.5 (97.9)	98.4 (97.8)	98.4 (97.5)
**Redundancy**	22.6 (22.8)	22.3 (22.1)	7.5 (7.7)	3.9 (3.9)	1.6 (1.6)	1.6 (1.6)	1.6 (1.6)

*Values for the highest resolution shell are shown in parentheses.

†R_merge_ = Σ_i_ | I_i_−〈I〉 |/Σ 〈I〉, where I is an individual intensity measurement and 〈I〉 is the average intensity for this reflection with summation over all data.

**Figure 1:**
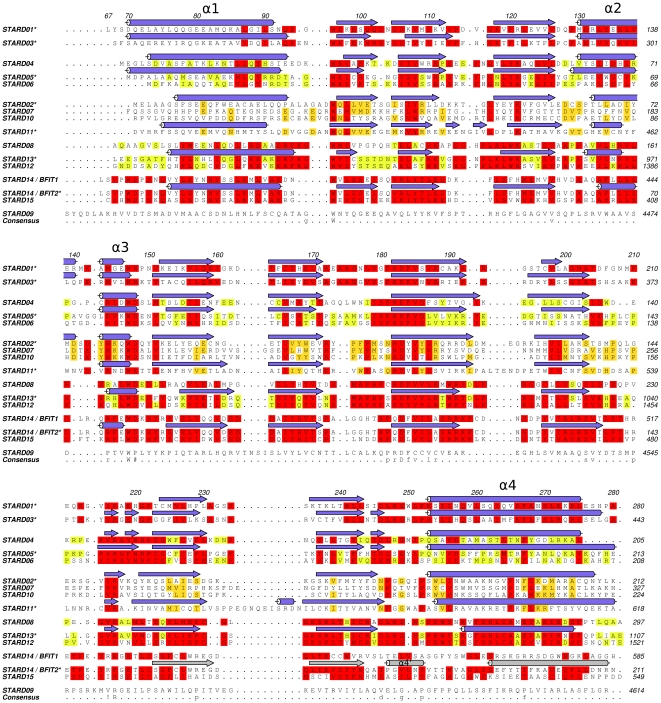
Structure based sequence alignment illustrating sequence conservation among human STARD proteins. Protein sequences were aligned based on the available crystal structures as detailed in Materials and Methods. Secondary structure elements are shown on top of each sequence for which a crystal structure is available, and α-helices are numbered. Secondary structural elements in the C-terminal section of STARD14 isoform are shown in grey to indicate their divergence. Asterisks following protein names indicate that crystal structures of human proteins are available.

**Figure 2:**
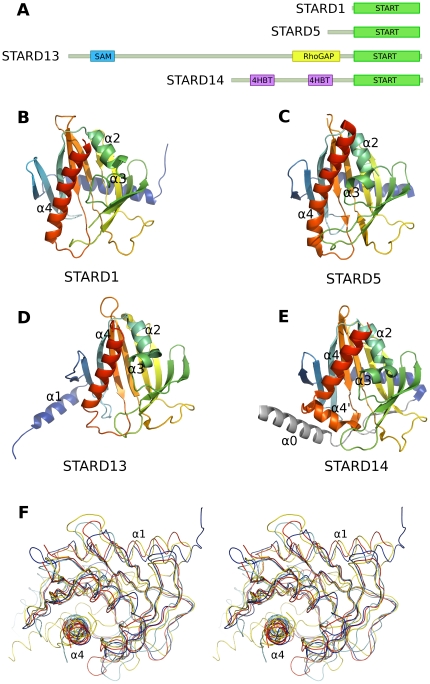
Overview of the crystal structures reported in this study. (A) Cartoon of the START domains studied here in context of the respective full-length proteins (drawn approximately to scale). (B–E) Side-by-side comparison of human STARD1, -5, -13, and -14 in a similar orientation. All START domain structures are colored from the N-terminus (blue) to the C-terminus (red) and the linker to the N-terminal thioesterase domain of STARD14 (panel E) is shown in grey. (F) Stereo view of a superposition of the backbone traces of the four crystal structures shown in panels B through E (blue, STARD1; red, STARD5; cyan, STARD13; yellow, STARD14). The view is that of panels B–E with an approximately 90° rotation downward toward the viewer.

Despite the low sequence identity among the START domains (Fig. 1) all structures show a conserved domain structure consisting of an α/β “helix-grip”: A curved antiparallel β-sheet on which two helices near the N- and C-terminus are packed forming a cavity to the concave side of the sheet (Fig. 2 and Datapack S1). The backbone atoms of all four proteins superimpose with an rmsd of 2.3 Å over 154 residues (Fig. 2F), and the backbone atoms of all START domains included in Table 1 superimpose with an rmsd of 2.3 Å over 138 residues.

### STARD1

**Figure 3:**
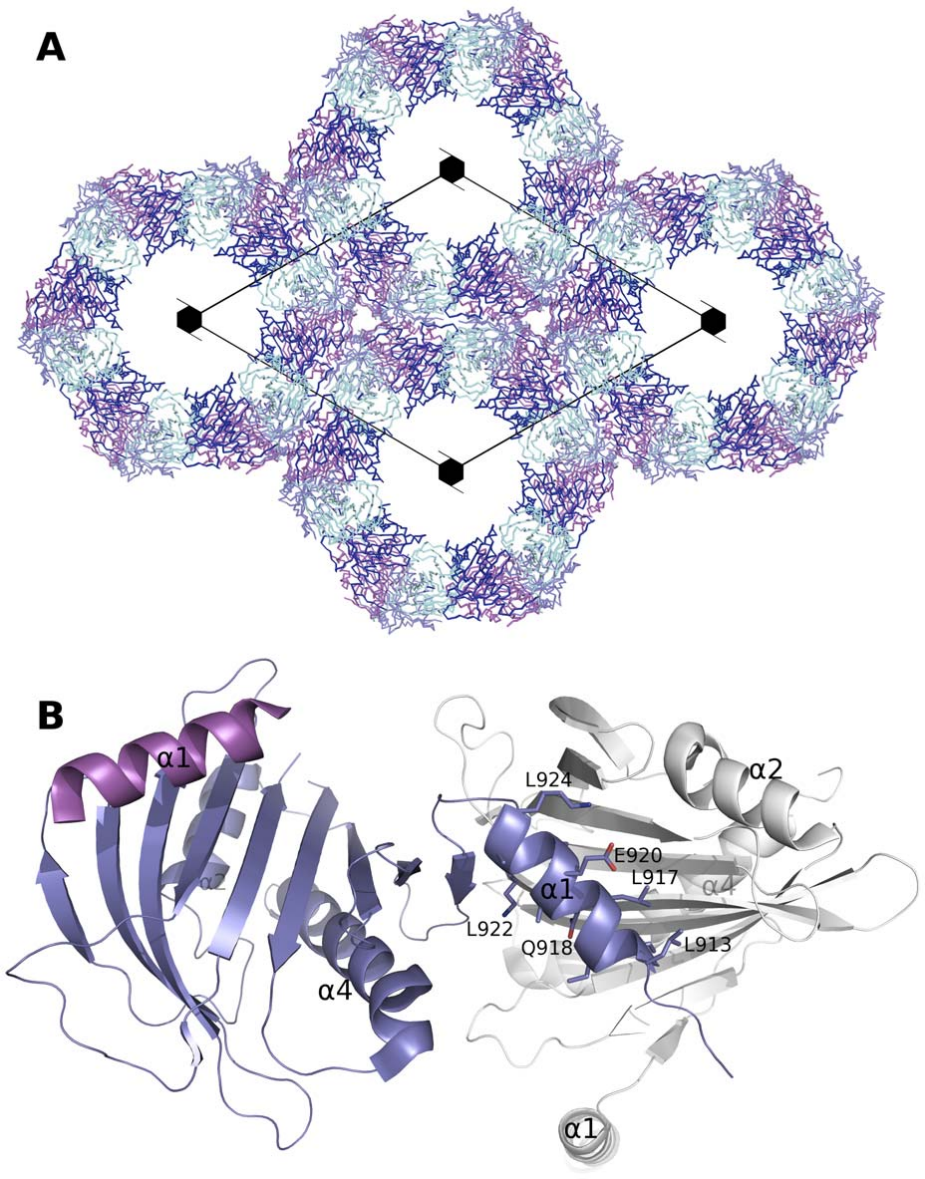
Notable properties of the STARD1 and STARD13 crystals. (A) Packing of STARD1 in the crystal lattice with the tube formed around the 6_3_-axis. Monomers A–D in the asymmetric unit are colored individually, and symmetry generated molecules around the axis are shown. (B) STARD13 structure displaying the N-terminal helix swap with the adjacent protein molecule in the crystal. Two monomers (blue and white) are shown and the N-terminal helix of a third monomer is shown in magenta. Side chains are displayed for one of the C-terminal helices.

We solved the crystal structure of STARD1, a member of the StAR group, at a relatively low resolution of 3.4 Å (Fig. 2B). Attempts to find better crystals were unfruitful, presumably due to the intriguing packing inside the lattice of these crystals: The asymmetric unit consists of four molecules that are organized as a long tube along the 6_3_ axis (Fig. 3A). The inside diameter of the tube is 75 Å, resulting in a solvent content of 60%.

Homology modeling and subsequent ligand docking trials were previously studied in an effort to understand biological functions of STARD1 [10]. The STARD1 crystal structure supports the homology model (PDB 2I93). Superposition of the crystal structure with the lowest energy homology model yields an rmsd of 1.5 Å for 205 out of the 213 Cα atoms. Major differences between these structures are found in the loops 191–196 and 209–215.

Cholesterol was included in the crystallization buffer. However, additional density which was observed in the cavity did not match the expected density of cholesterol. We believe that the cavity was either empty or partially occupied by a small ligand derived from the expression host or from the crystallization solution.

### STARD5

STARD5 [11], a member of the START only group (Table 1), binds specifically to cholesterol and 25-hydroxycholesterol. The closest homolog of human STARD5 with a crystal structure available in the PDB is mouse STARD4 [7], with 34% shared sequence identity between the proteins. We also used this structure of mouse STARD4 (PDB entry 1JSS) as a molecular replacement model. Alignment of the refined structure with mouse STARD4 gives an rmsd of 1.3 Å over 194 Cα-atoms. The STARD5 structure is naturally also closely related to other human START domains (Fig. 2).

### STARD13

The structure of human STARD13, a member of the RhoGAP group, is also most similar to mouse STARD4, with an rmsd of 1.8 Å for 164 Cα-atoms. The largest difference between STARD13 and other START domain structures lies in the N-terminal helix, which in STARD13 is swapped with the adjacent protein in the crystal (Fig. 2D and 3B). The swapped helix interacts with the expected area of the β-sheet, but runs in an opposite direction. This surprising helix-swapping may be an artifact of a truncated expression construct.

### STARD14

The asymmetric unit of the crystal of STARD14/ACOT11 contains a dimer. The large buried surface area between the monomers (900 Å^2^ per monomer, as determined by the PISA server [12]) indicates that this interaction could form also in solution. However, the full-length protein likely forms a trimer in the thioesterase domains of ACOT12 (PDB id. 3B7K), and the dimer interface of truncated STARD14 may only be a part of the biologically relevant assembly. The STARD14 structure differs slightly from the other START domain structures in that the C-terminal consensus α-helix is broken into two shorter helices (Fig. 1 and 2E).

The unique N-terminal helix (α0) of STARD14 (Fig. 2E) acts as a linker to the thioesterase domains. Interestingly, this N-terminal helix packs onto the C-terminal START domain helix (α4′) that is thought to undergo a conformational change upon ligand binding [13]. Based on the crystal structure it is feasible that the N-terminal helix upon ligand binding transmits a conformational signal to the thioesterase domains to regulate its activity. Thus, our results form a structural basis for interpreting the conformation of the N-terminal helix. This however requires verification by experiments with the full-length protein.

### Family wide structural comparison

Human START domains share a significant but low sequence identity (as low as 14%). As a consequence, homology-based sequence alignment methods make prediction of the positions of critical residues within the physiological START domain structures challenging. We generated a structure based sequence alignment by superposing all known START domain structures, and using this 3D alignment as a basis for aligning the sequences of the human START domain classes. This method yielded an improved alignment, and displayed similarities between individual proteins that have been overlooked by homology based methods (Fig. 1). When compared to previous family wide alignments [11] it is evident that the structure-based alignment has the similar overall features. It does not contain gaps within the secondary structure elements thus providing better alignment when the structure, but not necessarily the sequence, is conserved. On the other hand our structure-based alignment could be misleading for surface residues that are affected by crystal contacts, in particular for less well conserved loop residues of low structural importance. These regions often contain gaps in the alignment.

**Figure 4:**
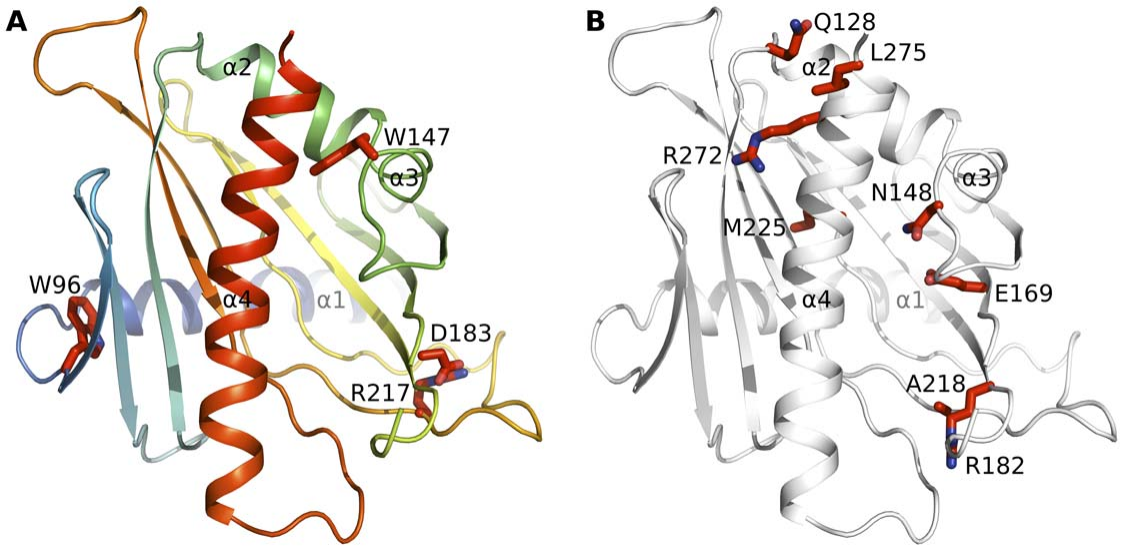
Sequence conservation among human STARD proteins. (A) Positions of strictly conserved residues, as identified by the alignment shown in Figure 1, mapped onto the structure of STARD1 (pdb entry 3P0L). (B) Residues mutated in CAH shown as side chains in the STARD1 structure.

Notably, there are three absolutely conserved residues (Trp96, Trp147 and Arg217; STARD1 numbering) and a highly conserved Asp183 that is replaced by the similar glutamate only in STARD4 (Fig. 1). Trp96, Asp183 and Arg217 are all on the “back” face of the β-sheet (Fig. 4A): Asp183 and Arg217 form a salt bridge, whereas Trp96 appears to be structurally important in aligning the N-terminal helix onto the β-sheet. Trp147 is likely of functional importance, specifically as a possible gate keeper in lipid ligand loading. It is located in a helical loop region and interacts with the C-terminal helix. In STARD1, the hydrophobic cluster around this residue has been proposed to stabilize the C-terminal helix in a closed conformation [14]. Conservation of this structural feature across the domain family indicates that a lipid binding mechanism via local unfolding or a significant conformational change in the C-terminal helix could be a family wide phenomenon. Mutation in the adjacent, highly conserved residue Asn148 has been observed in congenital lipoid adrenal hyperplasia (lipoid CAH) [15], which add further evidence to the functional importance of this region (Fig. 4B).

Lipoid CAH is linked also to other mutations in the STARD1 encoding gene. Some of these mutations lead to premature stop codons, while others change the protein activities and lipid binding capabilities [16], [15]. When the affected residues are mapped onto the STARD1 structure, it is evident that these changes occur in structurally important residues (Fig. 4B). However, with the exception of Asn148, the affected residues are not conserved across the family (Fig. 1). Most of the point mutations are in the C-terminal helix lining the ligand binding cleft or in residues interacting with this helix. These mutations would therefore cause changes in the dynamics of the ligand binding. It has been suggested that the C-terminal helix would undergo unfolding during ligand binding, and this suggestion is supported by the effects of the lipoid CAH mutations near the C-terminal helix [14]. The model of helix unfolding during cholesterol binding has been recently reviewed [17]. Some residues mutated in lipoid CAH are surface exposed indicating that they may change other interactions of the protein molecule as suggested for a gain of function mutation Q128R [15]. Also R182L is able to bind cholesterol but does not have “star-like activity” [18].

**Figure 5:**
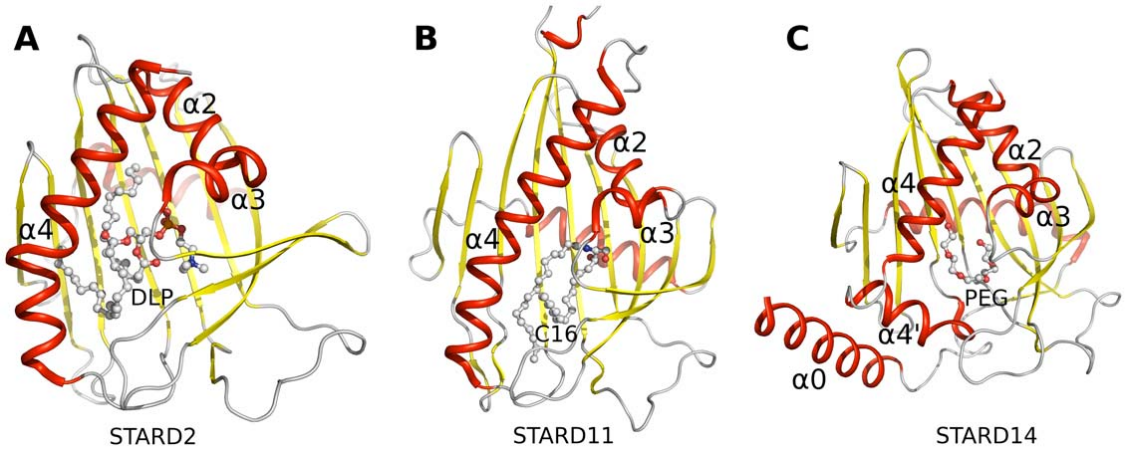
Ligand binding in START domains. Side-by-side comparison of the structures that have been solved with a ligand bound in the cavity: (A) STARD2 (1LN1) with 1,2-dilinoleoyl-SN-glycero-3-phosphocholine (DLP); (B) STARD11 (2E3R) C16-ceramide; (C) STARD14 (3FO5) with PEG or putative fatty acid.

Cavity sizes in the known START proteins vary from 873 Å^3^ to 2297 Å^3^ (based on the molecular surfaces of ligand bound as well as ligand free structures). STARD14 has clearly smallest cavity of the family. Cholesterol binding START domains have cavity sizes of 1014–1122 Å^3^, which is close to the size of the natural ligand. The largest cavity is observed for STARD2, which also binds larger ligand than other characterized members of the family (Table 1, Fig. 5). It is possible that the shape of the cavity changes upon ligand binding and therefore the size of the cavity is not directly related to the size of the ligands. However, together with a structure based sequence alignment, the cavity sizes suggest key residues and structural determinants of ligand binding and selectivity.

### StAR group - STARD1

Inspection of the ligand cavity of ligand free STARD1 suggests Glu169, Arg188, Leu199 and His220 as key residues in cholesterol binding. These side chains will likely change conformation upon ligand binding. Notably only His220 is conserved among the cholesterol binding members. Ligand docking predicted cholesterol binding to STARD1 involves a hydrogen bond between the cholesterol hydroxyl and either the Arg188 side chain or the backbone carbonyl of Leu199 [10]. Either of these ligand binding modes is consistent with the present STARD1 crystal structure.

### START only - STARD5

**Figure 6:**
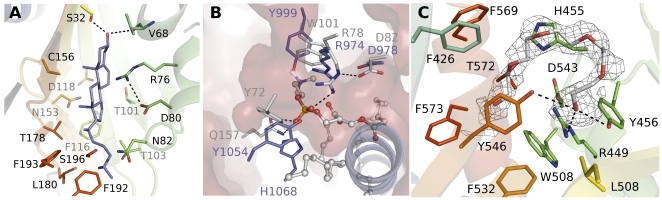
Ligand binding cavity of STARD5, -13, and -14. (A) Model of cholesterol binding to STARD5. (B) Lipid binding cavity of STARD13, with the cavity inner surface indicated in the background (magenta). Side chains that are conserved and structurally complementary among STARD13 (blue) and STARD2 (grey) are shown as sticks. The C-terminal helix of STARD13 is shown as a blue cartoon to illustrate clashes with the STARD2 ligand. (C) Ligand binding to STARD14. Difference density is contoured at 2σ around the modeled PEG molecule in monomer B to show the elongated shape with density for the head group resembling a carboxyl group.

In order to understand ligand binding in STARD5, we docked a cholesterol molecule to the binding cavity of the STARD5 structure. All the top ranked binding modes had cholesterol in the so-called “IN” conformation, with the hydroxyl group of cholesterol pointing towards the cavity (Fig. 6A). The binding mode is similar to the one predicted for other START domains [10]. In this scenario, the Ser132^199^ hydroxyl forms a hydrogen bond to the cholesterol hydroxyl in our best docking scenes as predicted for STARD3 (superscript numbering denotes positions in STARD1; see Fig. 1). A serine in this position is conserved in the cholesterol binding STARD3, -4 and -5, and there is a serine residue in the adjacent position in STARD6 that might fulfill the same function (Fig. 1). In all other START domain subfamilies there are hydrophobic residues at this position. Despite the conservation of this serine side chain within the cholesterol binding subclass, there is no similar serine in STARD1. Thus, in the absence of a START domain-cholesterol complex structure, the accurate binding mode of cholesterol can not be resolved.

STARD5, in contrast to STARD1, can also bind 25-hydroxycholesterol [19]. The crystal structure and docking model suggests a structural basis for binding specificity towards this ligand: The additional hydroxyl group is attached to a flexible hydrophobic tail of cholesterol, and this hydroxyl could be positioned within hydrogen bonding distance of the side chain of Thr103^171^ in STARD5 (Fig. 6A). In STARD1 the corresponding residue is alanine and together with the missing serine side chain (Ser132^199^) at the bottom of the cavity this could cause different ligand binding modes in STARD1 and STARD5, as discussed above.

### RhoGAP group - STARD13

The natural ligand of STARD13 is unknown. We looked to identify possible ligands based on the STARD13 side chains at the positions that correspond to those involved in lipid binding in other family members. From the crystal complexes of STARD11 and ceramides we know that Arg442^144^ and Glu446^148^ are the only conserved residues between the proteins making contacts with ceramide, Glu446^148^ being the most critical [8]. Notably, the STARD13 cavity also contains more polar side chains (three arginines, three histidines, an aspartate, a glutamate, two cystines and two tyrosines) compared to the cholesterol binding members, and the putative cholesterol hydroxyl binding Ser132^199^ of START5 is not conserved. The STARD13 cavity shares some characteristics with the members of the thioesterase group (discussed below). Intriguingly, some of the side chains that are involved in the interaction of STARD2 with dilinoleoylphosphatidylcholine are conserved in STARD13 (Fig. 6B): Arg974^144^ and Asp978^148^ are conserved in the corresponding position; Tyr999^169^ replaces Trp101^169^, Tyr1054^225^ replaces Tyr72^137^ and His1068^241^ replaces Gln157^223^. These side chains are also conserved in STARD8 of the same group, but not all in STARD12 (Fig. 1). Together, these properties indicate that STARD13 may bind a charged lipid.

Notably, the ligand binding cavity of STARD13 is smaller than that of STARD2 and elongated, with a small maximum diameter (Fig. 6B). This causes clashes between phosphatidylcholine and the C-terminal helix of STARD13 when the two structures are superposed. Upon ligand binding, the STARD13 cavity could expand due to movement of the C-terminal helix. The crystal structure however indicates that the natural ligand would be small – perhaps a fatty acid as proposed here for STARD14.

### Thioesterase group – STARD14

The lipid binding cavity of STARD14 is rather hydrophobic as it is lined by phenylalanine, valine, leucine and isoleucine side chains. The cavity also contains patches of charged and hydrophilic residues, possibly making specific interactions with an unknown ligand. Inside the STARD14 cavity we observed a continuous electron density that by its shape resembles a fatty acid (Fig. 6C). In the monomer B of the asymmetric unit the density was more continuous. In addition to the STARD14 model discussed here, the fatty acid-like density was present also in two other data sets that originated from different STARD14 protein constructs crystallized in different space groups (not shown). Despite several attempts with different strategies we could not identify the ligand by mass spectrometry. Therefore we modeled the density as a polyethylene glycol (PEG) fragment in the published model. Nevertheless, we believe that the natural ligands of STARD14 isoforms may be fatty acids based on several lines of evidence: (i) The cavity and conserved residues lining it are not consistent with the known START domain ligands cholesterol, phosphatidylcholine or ceramides. (ii) As STARD14 also contains the acyl-CoA thioesterase domains, fatty acid binding to the START domain might be physiologically meaningful. (iii) The rat ortholog of STARD14 has acyl-CoA thioesterase activity with specificity towards medium to long-chain (C12–18) fatty acyl-CoA substrates [20] and the STARD14 structure appears compatible for binding fatty acids containing up to 18 carbon atoms.

The STARD14 structure is expanded in comparison to the empty START domain structures, although the C-terminal helices are in a similar position as the C-terminal helix of STARD2 solved in complex with phosphatidylcholine. Possibly BFIT1 and BFIT2, the isoforms of STARD14, could have different ligand specificity. The crystallized form (BFIT2) contains two helices at the C-terminus whereas BFIT1 probably has only one, as seen in other START domains (Fig. 2). Interestingly, BFIT2 is more similar to STARD15 than BFIT1 (Fig. 1). The residues that would bind the putative head group of the fatty acid, Arg449^144^ and Tyr546^241^, are conserved in STARD15. Tyr546^241^ is a tryptophan in BFIT1 and Tyr456^151^ is phenylalanine in STARD15 (Fig. 1 and 6C). Other interactions around the PEG molecule found in the structure do not appear to be strictly conserved; however if the ligand is a fatty acid, these interactions are likely not specific and the selectivity would be accomplished based on the shape of the cavity rather than by specific side chain interactions.

## Discussion

A structure based alignment reveals important features within the START domain subfamilies, and highlights critical conserved residues involved in ligand binding. Of particular interest in the scope of this paper are STARD13 and STARD14, the family members for which the ligands are not known. STARD13 Arg974/STARD14 Arg449^144^ is highly conserved (Fig. 1) and is likely a key residue binding negatively charged lipids such as phosphatidylcholine and fatty acids. This is complemented by a negatively charged residue at position 148. This is generally an aspartate, but in STARD11 it is the longer glutamate, which makes important hydrogen bonds to the bound ceramide. Notably, these residues are not conserved in the StAR nor in the STARD9 groups. In the StAR group there is a similar ion pair between the β–strands, namely, Glu169 and Arg188, which is not found in other family members that bind cholesterol. Arg188 has been suggested to bind to the hydroxyl group of cholesterol and it is also present in PCTP and RhoGAP groups. Trp147 is absolutely conserved across the family, and since the Trp147 side chain interacts with hydrophobic residues of the C-terminal helix in all the structures (Fig. 4), this region is likely important for lipid access to the cavity. Our structural analysis also suggests that the cavities can adjust to binding several types of lipids due to their flexibility and hydrophobic nature, while the small differences in the key conserved residues make them specific towards different lipids. The third helix is likely also important for the lipid binding mechanism as it interacts with the C-terminal helix proposed to partially unfold during lipid binding.

### Conclusions

The crystal structures reported here help to gain a family wide understanding of the structural determinants within the START domain family. Use of these results to create a structure-based alignment helped to determine the conserved features within the family which are overlooked by sequence homology based methods. Many human START domains have unknown functions and their apo-structures form a structural basis for ligand identification thereby providing new leads to biological functions. All structures reported are relevant to disease. They are down- (STARD13) or up-regulated (STARD5) in cancers, mutations in them result in metabolic disorders (STARD1) or they are linked to obesity (STARD14; [21]). Based on our structural analysis we propose charged lipids as ligands for STARD13 and fatty acids as ligands for STARD14.

## Materials and Methods

### Cloning

The cDNAs coding for full-length human STARD1, STARD5, and STARD14 were obtained from the Mammalian Gene Collection (accession codes BC010550, BC004365 and BC093846, respectively). The cDNA encoding full-length human STARD13 was PCR amplified from pooled human brain, liver, placenta, and thymus cDNA libraries (Ambion). The sequence coding for residues STARD1^T66-R284^, STARD5^A6-E213^, STARD13^E51-I264^, and STARD14^R339-L594^ were subcloned into expression vector pNIC-Bsa4 by ligation-independent cloning. The resulting expression constructs contained a hexahistidine tag and a TEV-protease cleavage site (MHHHHHHSSGVDLGTENLYFQS) at the N-terminus.

### Protein expression and purification

Each expression construct was transformed into *E. coli* strain BL21(DE3)R3 pRARE (Novagen). Cultivation was done in a LEX large-scale expression system (Harbinger Biotechnology & Engineering). Cells were grown in Terrific Broth supplemented with 8 g/l of glycerol and 100 µl/l BREOX antifoam agent at 37°C. At an OD_600 nm_ of between 1 and 2 the temperature was lowered to 18°C, recombinant protein production was induced by addition of 0.5 mM isopropyl-β-d-thiogalactopyranoside, and cell growth was continued for 18 h. Cells were harvested by centrifugation and resuspended in 1.5 ml of buffer 1 per gram of wet cells (30 or 50 mM HEPES pH 7.5, 500 mM NaCl, 10% glycerol, 10 mM imidazole, 0.5 mM TCEP). Before lysis, 4 µl (1000 U) of Benzonase (Novagen) and one tablet of Complete EDTA-free protease inhibitor (Roche Biosciences) were added per 50 ml cell suspension, and cells were lysed by a freeze-thaw cycle and sonication. Cell debris was removed by centrifugation and the soluble fractions were filtered through a syringe filter (0.45 µm pore size). Cleared cell lysates were passed over 1-ml HiTrap Chelating columns (GE Healthcare) pre-equilibrated with buffer 1. The columns were washed sequentially with buffer 1 and buffer 1 containing 25 mM imidazole. Bound protein was eluted with buffer 1 containing 500 mM imidazole and loaded onto 16/60 HiLoad Superdex-75 columns (GE Healthcare). Gel filtration was performed in buffer 2 (30 mM HEPES or 30 mM sodium phosphate, pH 7.5, 300 mM NaCl, 10% glycerol, 0.5 mM TCEP). Fractions were pooled based on gel filtration profiles and purity determined by SDS-PAGE and Coomassie staining. STARD1 and STARD14 proteins were liberated from the hexahistidine tag by incubation with His6-tagged TEV-protease (20∶1 molar ratio) over night at room temperature and subsequent passage over 1-ml HiTrap Chelating columns. The proteins were concentrated to 28.2 mg/ml (STARD1), 21.8 mg/ml (STARD5), 4.3 mg/ml (STARD13), and 11.3 mg/ml (STARD14) using spin concentrators. TCEP was added to a final concentration of 2 mM and aliquots were flash-frozen and stored at −80°C. Proteins were typically more than 90% pure judged by SDS-PAGE analysis. Protein construct masses were verified by TOF-MS analysis.

### Crystallization and data collection

Crystallization was done by the sitting or hanging drop vapor diffusion method. Proteins in gel filtration buffer were mixed with reservoir solution (see Table 2 for details). For data collection crystals were briefly dipped in cryo solution supplemented with suitable additives (Table 2) and flash-frozen in liquid nitrogen.

Synchrotron radiation datasets were collected at ESRF, Grenoble, France and at BESSY, Berlin, Germany. Data sets were indexed, scaled, and reduced using XDS (Table 3) [22].

### Structure solution

Details of the structures are given in Table 4. The STARD1 structure was solved by molecular replacement with MOLREP included in the CCP4 suite [23] using the STARD3 (1EM2) structure as a model. MOLREP placed the first two molecules in the asymmetric unit and a third one after refinement. The fourth monomer was placed manually, using a helix density as guide, followed by a rigid body refinement in PHENIX [24]. A test set of reflections for STARD1 was selected with PHENIX to prevent creation of a biased set due to twinning. Twin refinement with operator h,-h-k,-l was done with PHENIX and the refined twin fraction was 0.149. This is in agreement with the estimated twin fraction using the diffraction data only (0.130). Model building was done with COOT [25].

**Table 4:** Refinement statistics.

Protein	STARD1	STARD5	STARD13	STARD14
**PDB entry**	3P0L	2R55	2PSO	3FO5
**Search model**	1EM2	1JSS	1JSS	-
**Ligand**	-	-	-	PEG
**R_work_** † **/R_free_** ‡	0.257/0.287	0.232/0.276	0.210/0.244	0.202/0.251
**Molecules/a.u.**	4	2	3	2
**No. atoms**				
**Protein**	6373	3292	4477	3813
**Ligands**	-	-	-	56
**Water**	-	26	-	168
***B*** **-factors (Å^2^)**				
**Protein**	128	78.8	90.1	39.6
**Ligands**	-	-	-	40.08
**Water**	-	51.1	-	28.5
**R.m.s deviations**				
**Bond lengths (Å)**	0.002	0.012	0.011	0.013
**Bond angles (°)**	0.512	1.375	1.454	1.386
**Ramachandran plot (%)**				
**Favored regions**	92.3	93.6	90.1	98.7
**Additionally allowed regions**	7.7	6.4	9.0	1.3
**Outliers**	-	-	0.9	-

†R_work_ is defined as Σ ||F_obs_ |−|F_calc_ || Σ | F_obs_ |, where F_obs_ and F_calc_ are observed and calculated structure-factor amplitudes, respectively.

‡R_free_ is the R factor for the test set (5–10% of the data).

The STARD5 structure was solved by molecular replacement with MOLREP [26] using pdb entry 1JSS as a model. The structure was refined initially with PHENIX and in the final stages with REFMAC5. TLS model consisting of 3 groups per monomer was used based on the suggestion by the TLSMD server [27].

STARD13 was solved by molecular replacement with MRBUMP [28] using pdb entry 1JSS as a model. The model was edited with CHAINSAW [29] and the best solution, with two monomers in the asymmetric unit, was found with PHASER [30]. After rigorous editing and refinement with PHENIX a third molecule was located with MOLREP using partially refined monomer as an input. Model building was done with COOT and REFMAC5 was used in the final refinement cycles.

The STARD14 structure was solved using Solve [31] with the three-wavelength MAD data. Resolve [32] was used to build the initial model, which was then improved by cycles of manual editing and PHENIX autobuild. The asymmetric unit consisted of two chains and one of them was used as a model in MOLREP using the native data. The model was further improved by 3 rounds of manual model building and automated building with arp-warp [33]. Final refinement cycles were done with REFMAC5.

### Analysis of structures and visualization

Models were validated with Molprobity [34]. Initial structure based sequence alignments were made with MultiProt [35] and Staccato [36] using PDB entries 3P0L, 1LN1, 1EM2, 1JSS, 2R55, 2E30, 2PSO and 3FO5. The alignment was edited with Bodil [37] (similarity matrix STRMAT110; minor manual edits) to include full domain sequences, and visualized with Aline [38]. Cavity sizes were calculated with CASTp [39]. The enhanced version of the article was prepared with ICM (Molsoft).

### Modelling

Docking of cholesterol to STARD5 structure was done with ICM (Molsoft). Residues surrounding the cavity were selected to indicate the binding site and initial docking of cholesterol was done keeping the residues fixed. Best conformations were energy minimized with ICM and the residues around the docked ligand were optimized.

## Supporting Information

Datapack S1 **Standalone iSee datapack - contains the enhanced version of this article for use offline. This file can be opened using free software available for download at** http://www.molsoft.com/icm_browser.html **.**
(ICB)

Text S1 **Instructions for installation and use of the required web plugin (to access the online enhanced version of this article).**
(PDF)
